# (+)-Clausenamide protects against drug-induced liver injury by inhibiting hepatocyte ferroptosis

**DOI:** 10.1038/s41419-020-02961-5

**Published:** 2020-09-19

**Authors:** Min Wang, Chun-Yu Liu, Tian Wang, Hong-Min Yu, Shu-Hua Ouyang, Yan-Ping Wu, Hai-Biao Gong, Xiao-Hui Ma, Gen-Long Jiao, Lei-Lei Fu, Qiong-Shi Wu, Hiroshi Kurihara, Yi-Fang Li, Tao Shen, Rong-Rong He

**Affiliations:** 1 Guangdong Engineering Research Center of Chinese Medicine & Disease Susceptibility, Jinan University, 510632 Guangzhou, China; 2 Department of Pharmacy, Hainan General Hospital (Hainan Affiliated Hospital of Hainan Medical University), 570311 Haikou, Hainan China; 3 International Cooperative Laboratory of Traditional Chinese Medicine Modernization and Innovative Drug Development of Chinese Ministry of Education (MOE), College of Pharmacy, Jinan University, 510632 Guangzhou, China; 4 Guangdong Province Key Laboratory of Pharmacodynamic Constituents of TCM and New Drugs Research, College of Pharmacy, Jinan University, 510632 Guangzhou, China; 5 Key Lab of Chemical Biology (MOE), School of Pharmaceutical Sciences, Shandong University, 250012 Jinan, China; 6 The Spine Surgery Department, The First Affiliated Hospital of Jinan University, 510632 Guangzhou, China; 7 School of Life Science and Engineering, Southwest Jiaotong University, 610031 Chengdu, China

**Keywords:** Cell death, Pharmacology

## Abstract

Drug-induced liver injury is the major cause of acute liver failure. However, the underlying mechanisms seem to be multifaceted and remain poorly understood, resulting in few effective therapies. Here, we report a novel mechanism that contributes to acetaminophen-induced hepatotoxicity through the induction of ferroptosis, a distinctive form of programmed cell death. We subsequently identified therapies protective against acetaminophen-induced liver damage and found that (+)-clausenamide ((+)-CLA), an active alkaloid isolated from the leaves of *Clausena lansium* (Lour.) Skeels, inhibited acetaminophen-induced hepatocyte ferroptosis both in vivo and in vitro. Consistently, (+)-CLA significantly alleviated acetaminophen-induced or erastin-induced hepatic pathological damages, hepatic dysfunctions and excessive production of lipid peroxidation both in cultured hepatic cell lines and mouse liver. Furthermore, treatment with (+)-CLA reduced the mRNA level of prostaglandin endoperoxide synthase 2 while it increased the protein level of glutathione peroxidase 4 in hepatocytes and mouse liver, confirming that the inhibition of ferroptosis contributes to the protective effect of (+)-CLA on drug-induced liver damage. We further revealed that (+)-CLA specifically reacted with the Cys-151 residue of Keap1, which blocked Nrf2 ubiquitylation and resulted in an increased Nrf2 stability, thereby leading to the activation of the Keap1–Nrf2 pathway to prevent drug-induced hepatocyte ferroptosis. Our studies illustrate the innovative mechanisms of acetaminophen-induced liver damage and present a novel intervention strategy to treat drug overdose by using (+)-CLA.

## Introduction

Drug-induced liver injury (DILI) is a major cause of acute liver and kidney failures. The yearly incidence rate of DILI is between 13.9 and 19.1 per 100,000 individuals, and approximately 20% of children who suffer from DILI will develop liver failure^1^. Acetaminophen (APAP) overdose is the predominant cause of DILI, and as such its underlying mechanism has been heavily investigated using cell lines and animal models^2,3^. It is now well-accepted that APAP can induce hepatoxicity through two common forms of cell death, namely apoptosis and necrosis^4^. Notably and intriguingly, a recent study indicated that the third form of cell death, termed ferroptosis, might also contribute to APAP-induced DILI in vitro^5,6^. However, in-depth studies are required to define the key role of ferroptosis in DILI, which will open a new door for developing effective intervention strategies.

Ferroptosis, a form of programmed oxidative cell death, differs from other cell death forms at the morphological, biochemical, and genetic levels. The occurrence and execution of ferroptosis have been reported to be regulated by amino acids, lipids, and iron metabolism^7^. For example, a classic ferroptosis inducer, erastin, inhibits system Xc^−^, resulting in suppression of cellular cysteine uptake and depletion of glutathione (GSH). The latter participates in the main defense mechanisms of antioxidant against reactive oxygen species (ROS). Glutathione peroxidase 4 (GPX4) uses GSH to repair lipids and converts toxic lipid hydroperoxides into non-toxic lipid alcohols^8^. Depletion of GSH inactivates GPX4, leading to overwhelming lipid peroxidation, ultimately the induction of ferroptosis. Ferroptotic cell death can be blocked by iron chelators, lipophilic antioxidants, and lipid peroxidation inhibitors like ferrostatin-1 (fer-1)^7,9^.

The Keap1–Nrf2 pathway, an essential regulator of oxidative stress, has been recently discovered to participate in protecting cells against ferroptosis^10^. Under unstressed conditions, nuclear factor erythroid 2-related factor 2 (Nrf2) is constantly ubiquitinated and degraded in proteasomes in a Keap1-dependant manner. In response to stress, the cysteine residues in Keap1 (Kelch-like ECH-associated protein 1) react with oxidants or electrophiles, leading to its modification and separation from Nrf2^11^. Subsequently, the released Nrf2 is translocated to the nucleus, forming heterodimers with small Maf proteins to bind to antioxidant response elements (ARE) in the promoter region of cytoprotective genes and to enhance their transcription^12^. Importantly, a growing body of evidence has indicated that Nrf2 plays crucial roles in both ferroptosis-related pathways, including lipid metabolism, iron homeostasis, and the pathogenesis of DILI^13–15^. Hence, the Keap1–Nrf2 pathway has been considered as a potential strategy for the treatment of DILI, and several natural products can inhibit DILI by modulating the Keap1–Nrf2 pathway in the liver^16–18^.

**Fig. 1:**
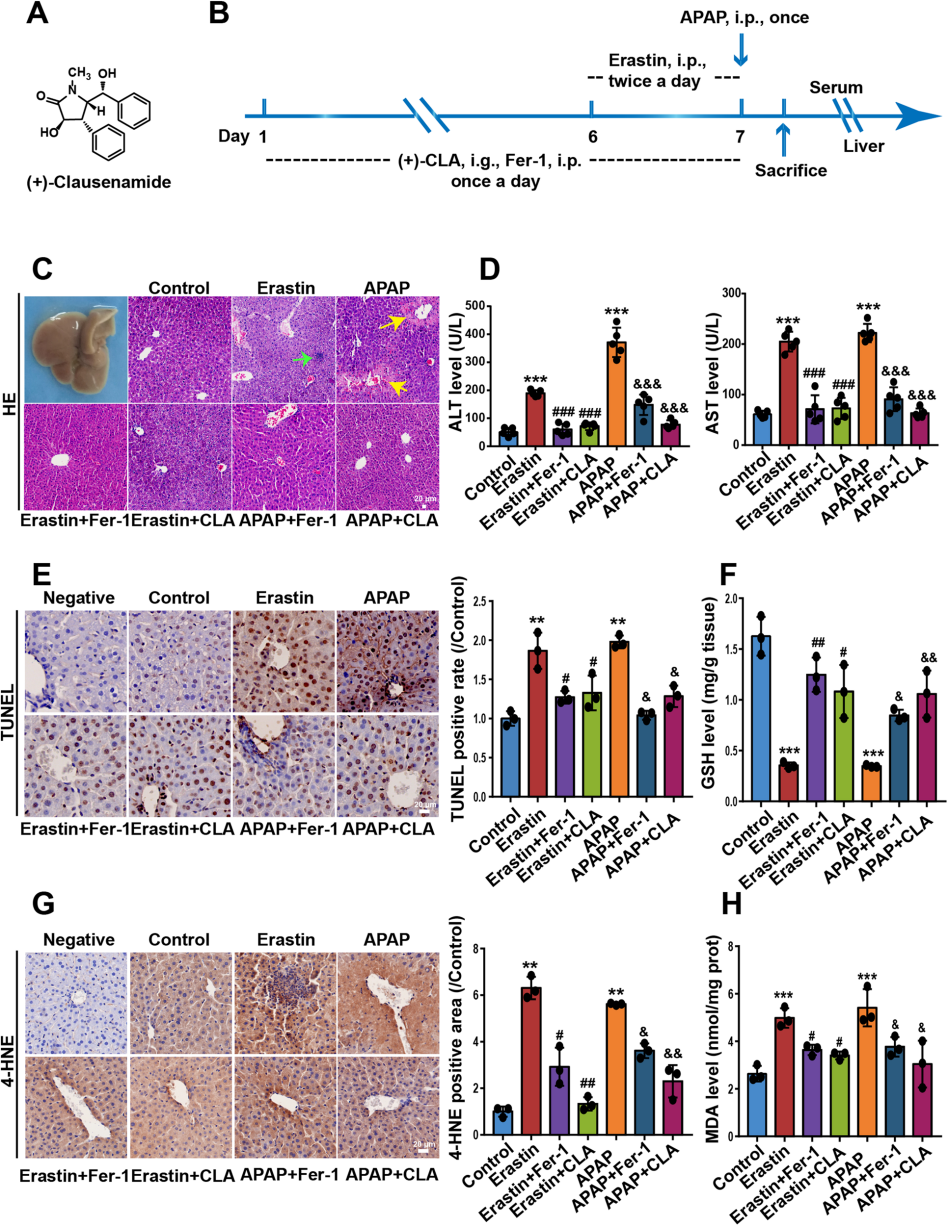
(+)-CLA protects against APAP- and erastin-induced liver lipid peroxidation in vivo. **a** The chemical structure of (+)-CLA. **b** Schematic diagram of the experimental procedures. **c** Histopathological changes were examined by H&E staining and observed with microscopy. The yellow and green arrows indicate bleeding and inflammatory infiltration, respectively. **d** Serum levels of ALT and AST were detected by commercial assay kits. **e** The dead hepatocytes were monitored by TUNEL staining in fixed liver tissue sections. Representative images are shown in the left panel and the quantification of TUNEL positive cells is presented in the right panel. **f** The GSH content in liver tissue was assayed by HPLC-ECD. **g** 4-HNE protein expression measured by IHC analysis in fixed liver tissue sections. **h** The content of MDA in the liver tissues was evaluated by an MDA assay kit. Data are expressed as mean ± SD and the statistical differences were analyzed by one-way ANOVA (*n* ≥ 3). **P < 0.01, ***P < 0.001 vs. control group; ^#^*P* < 0.05, ^##^*P* < 0.01, ^###^*P* < 0.001 vs. erastin group; ^&^*P* < 0.05, ^&&^*P* < 0.01, ^&&&^*P* < 0.001 vs. APAP group.

Clausenamides (CLA), natural racemic pyrrolidone compounds, are isolated from the leaves of *Clausena lansium* (Lour.) Skeels, a popular fruit tree in southern China. The isolated compounds of *Clausena lansium* share a wide range of pharmacological activities, and CLA have been reported to protect against chemical-induced liver injury independently of its capability of scavenging hydroxyl radicals^19–21^. The enantiomer (+)-CLA (Fig. 1a) has the best effect on promoting the synthesis of GSH and enhancing the activity of glutathione S transferase (GST)^22^. We thus proposed that (+)-CLA might hold the potential to regulate hepatocyte ferroptosis to benefit DILI. In the present study, substantial in vivo and in vitro evidence proved that hepatocyte ferroptosis was engaged in APAP-induced DILI. Further data demonstrated (+)-CLA directly interacted with Keap1 at the Cys-151 residue to block the ubiquitin-mediated degradation of Nrf2, thus inhibited APAP-induced ferroptosis to ameliorate liver injury. This study provides the scientific basis for the research and development of hepatoprotective drugs targeting lipid peroxidation and ferroptosis.

## Materials and methods

### Chemicals and reagents

Erastin (S7242), fer-1 (S7243), necrostatin-1(nec-1, S8037), and ZVAD-fmk (S7023) were purchased from Selleck Ltd. (Shanghai, China). APAP (1003031), GSH (PHR1359), cycloheximide (CHX, 239763), dimethyl sulfoxide (DMSO, D2650), deferoxamine (DFO, D9533) and MG-132 (474790) were purchased from Sigma-Aldrich (Shanghai, China). (+)-CLA was supplied by the China Academy of Chinese Medical Science. Aminotransferase (ALT), aspartate aminotransferase (AST), malondialdehyde (MDA), and lactate dehydrogenase (LDH) detection kits were purchased from Nanjing Jiancheng Bioengineering Institute (Nanjing, China). Nicotinamide adenine dinucleotide phosphoric acid (NADPH) assay kit was purchased from Comin Biotechnology (Suzhou, China). Hematoxylin and eosin (H&E) staining kit, 4′,6′ diamidino-2-phenylindole (DAPI), Annexin V-FITC/PI apoptosis kit, and lysis buffer were purchased from Beyotime Technology (Shanghai, China). In situ cell death detection peroxidase dismutase (POD) kit was purchased from Roche (Germany). Pierce BCA protein assay kit and NE-PER™ nuclear and cytoplasmic extraction kit were purchased from Thermo Scientific (Shanghai, China). Trizol was purchased from Tiagen Biotechnology (Beijing, China). SYBR Green kit was bought from Transgen Biotechnology (Beijing, China). Primers for detecting Ptgs 2, GSTA1, GSTM2, heme oxygenase-1 (HO-1), glutamate-cysteine ligase (GCLM), NAD(P)H: quinone oxidoreductase 1 (NQO1), Nrf2, glucose transporter 1 (GLUT1), thioredoxin reductase 1 (TXNRD1), β-actin, and 18s mRNA were synthesized by Generay Biotechnology (Shanghai, China). Anti-Nrf2 antibodies were purchased from Abcam (ab62352) and Proteintech Group (16396-1-AP). Antibodies for solute carrier family 7 member 11 (SLC7A11, ab37185), GPX4 (ab125066), 4-hydroxynonenal (4-HNE, ab46545), and TATA binding protein (TBP) (ab125009) were purchased from Abcam (MA, USA). Anti-Keap1 antibody was purchased from Cell Signal Technology (MA, USA). Anti-glyceraldehyde 3-phosphate dehydrogenase (GAPDH) antibody (FD0063) and FDbio-Pico ECL kit were from Fude Biological Technology (Hangzhou, China). Alexa Flour 594 and antibodies for Keap1(10503-2-AP), GCLM (14241-1-AP), hemagglutinin (HA, 66006-2-Ig), IgG (B900610) and β-actin (66009-1-lg) were purchased from Proteintech Group (IL, USA). Antibodies for NQO1 (SC-32793) and HO-1 (SC-390991) were purchased from Santa Cruz Biotechnology (CA, USA). Phosphatidylcholine hydroperoxide (PCOOH) was obtained from the University of Pittsburgh. Liperfluo dye (L248) was purchased from Dojindo Molecular Technologies, Inc. (Tokyo, Japan). Boron-dipyrromethene (BODIPY) 581/591 C11 (D3861) and lipofectamine 2000 (11668019) were purchased from Life Technologies. Corp. (CA, USA).

### Animals and treatments

Male C57BL/6 mice aged 8–10 weeks were purchased from Guangdong Experimental Animal Center (Guangzhou, China). The animals were maintained on a 12 h light-dark cycle in a regulated temperature and humidity environment for 1 week before drug administration. Treatment protocols are shown in Fig. 1b. (+)-CLA (50 mg/kg/day, i.g.) or fer-1 (2.5 μmol/kg/day, i.p.)^21,23^ were administered for 7 consecutive days. To induce liver injury, mice were injected with erastin (100 mg/kg/day, i.p., twice a day) on both the 6th and 7th day, or a single dose of APAP (600 mg/kg/day, i.p.) on the 7th day after overnight food deprivation. The serum and livers were obtained for analysis. All the animal experiments were approved by humanistic animal care standards and authorized by the Laboratory Animal Ethics Committee of Jinan University. All animal protocols followed the guidelines for the Care and Use of Laboratory Animals which published by the US National Institutes of Health (NIH Publication No. 85–23, 1996).

### Determination of serum ALT and AST levels

Liver injury was analyzed by measuring the serum levels of ALT and AST. Serum samples were collected from blood after centrifugation at 1200 × *g* for 10 min. ALT and AST in the serum were detected using commercial assay kits under the guidance of the manufacturer’s instructions.

### H&E staining and immunohistochemical (IHC) analysis

The livers were chipped from mice at the same position and were fixed in 4% paraformaldehyde (PFA). PFA fixed tissues were embedded in paraffin and sections were sliced at 4.5 μm thickness and mounted on slides. H&E staining was used for morphological studies.

After deparaffinization with xylene and rehydration with gradient alcohol, the slices were boiled in 10 mM citrate buffer for 20 min for antigen retrieval. When the slices were cooled down, 0.1% Triton X-100 was used to permeabilize the cell membrane and 3% hydrogen peroxide was applied to quench endogenous peroxidase at room temperature for 10 min in the dark. Then the slices were incubated with anti-4-HNE antibody (rabbit, 1:200, Abcam) at 4 °C overnight in a humid cassette after blocking with goat serum for 1 h. Slices were washed with phosphate buffer saline (PBS, three times, 10 min) and incubated with biotinylated goat antirabbit secondary antibody for 1 h at room temperature. Biotin-streptavidin horseradish peroxidase (HRP) detection systems were used to detect immunoreactivity, then the sections were counterstained with hematoxylin and sealed with neutral resins.

### Terminal dexynucleotidyl transferase (TdT)-mediated dUTP nick end labeling (TUNEL) staining

For the detection of hepatic nuclear DNA strand breaks, an in situ cell death detection kit, POD was used to stain the paraffin-embedded sections according to the manufacturer’s instructions. The sections were counterstained with hematoxylin and sealed with neutral resins. TUNEL-positive cells which were characterized with brown nuclei were counted using image J software.

### Measurement of MDA, NADPH, and GSH contents

Hepatic MDA content was measured using an MDA assay kit. NADPH content was measured using a coenzyme NADPH II content kit. The GSH separation was achieved on a C18 column using 6% acetonitrile solution and detected by a CoulArray detector. The pH of the mobile phase was adjusted with phosphoric acid to 3.0 and the GSH samples were filtered through a 0.2 μm hydrophilic polypropylene membrane filter before analysis. GSH content was quantified based on a standard curve.

### Isolation of total RNA and quantitative real-time reverse transcription polymerase chain reaction (qRT-PCR)

Total RNA of liver tissue was isolated using Trizol and transcribed into cDNA with DNAase treatment. The purification of RNA was performed according to the ratio of absorbance at 260 and 280 nm. qRT-PCR was performed with SYBR Green kit. The relative gene expression was normalized by the comparative Ct (2^−△△Ct^) with 18S or β-actin gene expression. The specific primer sequences applied in this study are presented in supplementary table 1.

### Protein extraction and western blot analysis

Whole cell extracts from tissues or cells were extracted with cell lysis buffer. Cytosolic and nuclear proteins were isolated using a NE-PER^TM^ nuclear and cytoplasmic extraction kit according to the manufacturer’s instructions. Protein expression was analyzed by western blot. Proteins in liver tissues or cell lysates were electrophoretically separated by sodium dodecyl sulfate polyacrylamide gel electrophoresis (SDS-PAGE), transferred to a nitrocellulose membrane, and blocked with skimmed milk powder and incubated at 4 °C overnight with primary antibodies. The membranes were washed with tris buffered saline with tween 20 (TBST) and incubated with secondary antibodies conjugated with HRP. The protein bands were detected by ECL reagents using the Tanon system. Chemiluminescent signals were analyzed using the Image J analyze system.

### Determination of GPX4 enzymatic activity in liver tissue by liquid chromatograph-mass spectrometer (LC-MS)

Liver GPX4 samples were prepared as described^24^. In the test mixture, 10 μM of PCOOH, 200 μg of GPX4 samples and 5 mM GSH were added to a glass tube with 500 μL total volume using GPX4 reaction buffer (final buffer concentration of 25 mM sodium phosphate, 125 mM NaCl, 1 mM EDTA, 0.1 mM DFO, 0.1% Triton X-100; pH 8.0). Then the mixture was vortex mixed for 1 min and reacted for 10 min at 37 °C. Thereafter, it was extracted by a chloroform:methanol (2:1 vol/vol) solution three times. The chloroform layers were collected into another glass container, dried by nitrogen gas and re-dissolved in 100% methanol before LC-MS analysis.

LC-MS was carried out on an Ultimate 3000 Rapid Separation LC (Thermo Fisher Scientific) coupled to a quadrupole Orbitrap ion trap mass spectrometer. The lipid extract was separated on a UPLC HSS T3 C18 column (2.1 × 100 mm; 1.8 μm particle size) at 40 °C column temperature. The mobile phase was 2 mM ammonium formate and 0.1% formic acid in water (A) or methanol (B). The gradient curve was 75% B at 0 min, 100% B at 6 min, 100% B at 20 min, 75% B at 20.1 min, and 75% B at 22.5 min. The flow rate was 0.3 mL/min with an injection volume of 1 μL. The mass range between *m/z* 886.5809 ± 2 and 870.5860 ± 2 was recorded during the acquisition, and SIM of *m/z* 886.5809 and 870.5860 [M+HCOO]^−^ was analyzed to determine the amount of PCOOH and PCOH, respectively. The GPX4 enzymatic activity was expressed as the amount of PCOOH converted per mg of GPX4 samples within 30 min. The conversion rate (%) = (*T*_0_ PCOOH – *T*_30_ PCOOH)/*T*_0_ PCOOH × 100%.

### MTT cell viability and LDH release assays

Cells (5000 cells per well) were seeded in 96-well plates and pretreated with (+)-CLA, ZVAD-fmk, nec-1 and fer-1 for 1 h and then treated with APAP or erastin for 24 h. After treatment, cell viability was detected by MTT assay, and LDH release was determined using a LDH cytotoxicity detection kit according to the manufacturer’s instructions.

### BODIPY staining and confocal imaging

Hepa RG cells were plated in confocal dish at 37 °C and treated with or without (+)-CLA for 24 h. After treatment, the cells were washed three times with PBS for 30 min and fixed with 4% PFA for 15 min and permeabilized with 0.1% Triton-100 for 10 min at room temperature. Then the cells were incubated with BODIPY 581/591 C11 at 37 °C for 1 h. After washing, nucleus was stained with DAPI in PBS for 10 min. The confocal microscope images were obtained at 484/510 nm excitation/emission using a Carl Zeiss LSM 510 laser scanning confocal microscope (Tokyo, Japan).

### Liperfluo staining and flow cytometry analysis

Cells were plated in 100 mm dishes and treated with (+)-CLA for 24 h. After treatment, cells were washed and incubated with 20 μM liperfluo dye for 1 h at 37 °C. Cells were trypsinized, centrifuged, and resuspended in sheath before analysis.

### Co-immunoprecipitation (Co-IP) analysis

Cells were transfected with Nrf2 and Keap1 plasmids with a ratio of 1:1 using lipofectamine 2000, and cultured for 40 h. The transfected cells were treated with or without (+)-CLA for 6 h, and then were co-treated with 10 μM MG-132 for an additional 6 h. The cell lysates were diluted four-fold with the buffer including 1% Triton X-100, 150 mM NaCl, and 10 mM Tris-HCl. Protein A beads and anti-Keap1 antibodies were added for incubation at 4 °C overnight. Immunoprecipitated proteins were subjected to western blot analysis with Nrf2 and Keap1 antibodies.

### Nrf2 immunostaining (IF) and confocal imaging

Cells were seeded in dishes which had been pre-placed with glass covers and were treated with indicated doses of (+)-CLA for 18 h. Then, the glass covers were washed with PBS three times and fixed with −20 °C methanol/acetone (1:1) for 10 min. The glass covers were then incubated with anti-Nrf2 antibody at 4 °C overnight. After washing in PBS, the glass covers were incubated with Alexa Flour 594 and DAPI for the indicated time. An Olympus BX53 fluorescence microscope coupled to an Olympus DP73 digital camera (Tokyo, Japan) was employed to image the fluorescence signals.

### Ubiquitination detection

Cells were transfected with expression vectors for HA-ubiquitin, Nrf2, and Keap1. Then, the transfected cells were exposed to 10 μM MG-132 along with or without (+)-CLA for 6 h. Western blot analysis was used to analyze 20 μL cell lysates to evaluate the expression of Nrf2. The remaining cell lysates were diluted four-fold with the buffer including 1% Triton X-100, 150 mM NaCl, and 10 mM Tris-HCl. Then protein A beads, and anti-Nrf2 and anti-IgG antibodies were added at 4 °C overnight. Immunoprecipitated proteins were subjected to western blot analysis with anti-HA antibodies.

### Half-life measurement of Nrf2 protein

Cells were seeded in 35 mm dishes, and pre-incubated with or without (+)-CLA for 8 h. After exposure to 50 μM CHX, cells were collected at 0, 10, 20, 30, and 40 min. Western blot analysis was then used to analyze protein level of Nrf2. The intensities of Nrf2 bands were quantified using Image J analyze system and plotted against time following CHX treatment.

### Cellular thermal shift assay (CETSA)

For the cell lysate CETSA experiments, cells were collected and freeze-thawed three times using liquid nitrogen. The lysates were diluted with PBS and divided into two aliquots, with one aliquot being treated with (+)-CLA (20 μM) and the other aliquot as a control (DMSO). After 1 h incubation at 37 °C, the lysates were heated individually at different temperatures (42–62 °C) for 3 min followed by cooling for 3 min at room temperature. For the intact cell experiments, the cells treated with (+)-CLA (20 μM) for 24 h were heated as above described followed by lysis using three cycles of freeze-thawing with liquid nitrogen. All the samples were centrifuged at 16,600×*g* for 20 min at 4 °C to separate the supernatant and pellet. The soluble fractions were isolated and analyzed by western blot analysis.

### siRNA transfection

Cells were cultured in 60 mm dishes (for western blot analysis) or 96-well plates (for cell viability assay). Control siRNA (no silencing) and Nrf2 siRNA were transfected into cells using lipofectamine 2000.

### Annexin-V/PI staining and flow cytometry analysis

Annexin V-FITC/PI apoptosis kit was used to evaluate apoptotic ratio according the manufacturer’s instructions. Cells were pre-treated with or without ZVAD-fmk for 1 h, and then incubated with APAP for another 24 h. After treatment, cells were washed and stained with PI followed Annexin V-FITC at room temperature for 15 min. To quantify apoptotic ratio, cell samples were analyzed by a flow cytometry.

### Statistical analysis

Results were expressed as means ± SD. GraphPad Prism version 7.0 statistical software was used for statistical analysis. Multiple groups were compared by *t*-tests, one-way ANOVA analysis or two-way ANOVA and a probability (*P*) value < 0.05 was considered statistically significant.

## Results

### (+)-CLA protects against APAP- and erastin-induced liver injury through inhibiting lipid peroxidation in vivo

Mice were challenged with liver injury reagents according to the experimental procedures as depicted in Fig. 1b. Firstly, we determined the levels of serum aminotransferases, and examined the histopathological changes and lipid peroxidation levels in the liver tissue of APAP-treated mice. The serum levels of ALT and AST indicated that (+)-CLA at different doses (50 and 100 mg/kg) significantly improved liver functions of APAP-treated mice (Fig. 1d and Fig. S1). H&E staining (Fig. 1c and Fig. S2) and TUNEL staining (Fig. 1e) showed that (+)-CLA alleviated the hepatic histopathology and hepatic cell death of mice treated with APAP. In addition, (+)-CLA reversed the decrease of GSH content (Fig. 1f) and inhibited the production of MDA and 4-HNE, two lipid peroxidation markers in the livers of APAP-treated mice liver (Fig. 1g, h and Fig. S3). These results imply that lipid peroxidation is engaged in DILI, which might be involved in the hepatoprotective mechanism of (+)-CLA. To verify this finding, a lipid peroxidation/ferroptosis inducer erastin was employed to establish DILI. As expected, erastin injection caused abnormal liver function and structure, together with an accumulation of lipid peroxidation. Of note, (+)-CLA also showed a significant protection against erastin-induced liver injury and lipid peroxidation in mice (Fig. 1c–h**)**. The above results suggest that (+)-CLA attenuates DILI by inhibiting lipid peroxidation.

### (+)-CLA suppresses APAP-induced and erastin-induced hepatocyte ferroptosis in vivo

**Fig. 2:**
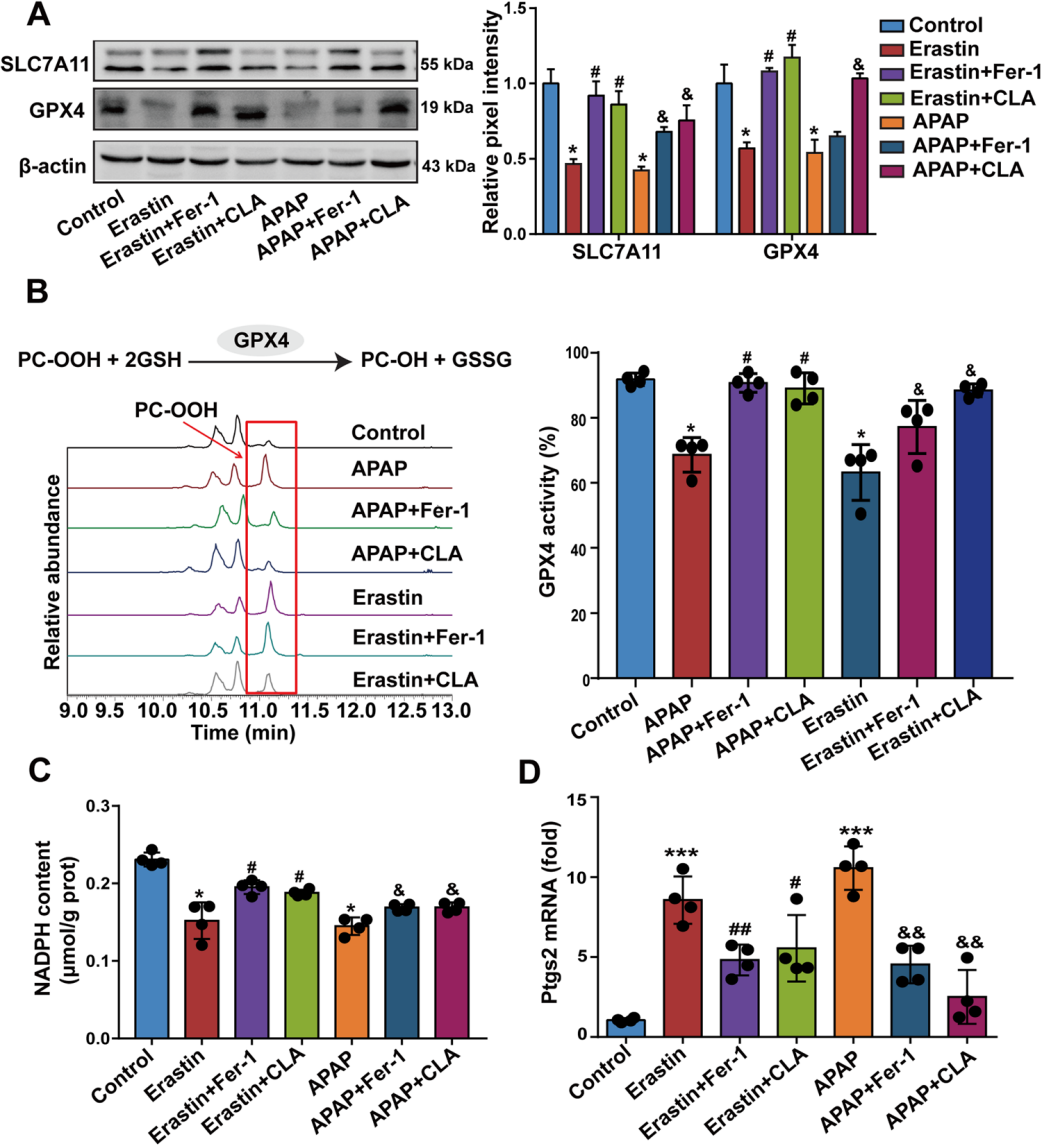
(+)-CLA inhibits APAP- and erastin-induced ferroptosis in vivo. **a** Protein levels of SLC7A11 and GPX4 in liver tissue were detected by western blot (left panel). The band intensities relative to β-actin were quantified by image J software (right panel). **b** GPX4 activity in liver tissue was determined by LC-MS using PCOOH as substrate (left panel) and expressed as PCOOH conversion rate (%) (right panel). **c** Hepatic NADPH content was assayed by an NADPH assay kit. **d** Ptgs2 mRNA levels were determined by qRT-PCR analysis. Data are expressed as mean ± SD and the statistical differences were analyzed by one-way or two-way ANOVA (**a**, two-way ANOVA; **b**–**d**, one-way ANOVA, *n* ≥ 3). *P < 0.05, ***P < 0.001 vs. control group; ^#^*P* < 0.05, ^##^*P* < 0.01 vs. erastin group; ^&^*P* < 0.05, ^&&^*P* < 0.01 vs. APAP group.

Lipid peroxidation can heavily disrupt cellular function, and lethal lipid ROS are the driving force of ferroptosis^25^. Our above discovery of lipid peroxidation in DILI prompted us to explore whether ferroptosis is involved in APAP-induced liver injury and the protective effects of (+)-CLA. We firstly examined the alterations of SLC7A11 and GPX4. These are, respectively, two key upstream and downstream regulators in the ferroptosis process^26^. Our data showed that both APAP and erastin decreased the protein levels of SLC7A11 and GPX4 (Fig. 2a) and increased the activity of the GPX4 enzyme (Fig. 2b) in mouse liver. In contrast, fer-1, a small-molecule inhibitor of ferroptosis, and (+)-CLA inhibited APAP-induced or erastin-induced changes in SLC7A11 and GPX4 (Fig. 2a, b). Further, we measured the level of NADPH, which has been implicated as a biomarker of ferroptosis sensitivity^27^, in liver tissue. Both fer-1 and (+)-CLA were found to recover NADPH content diminished by APAP or erastin treatment (Fig. 2c). In addition, we detected gene expression of Ptgs2, a recently identified standard of ferroptosis in vivo^8^. Results showed that fer-1 and (+)-CLA downregulated Ptgs2 mRNA levels in the livers of mice treated with APAP or erastin (Fig. 2d). The above data indicated an essential role of hepatocyte ferroptosis in DILI and the relation of the in vivo hepatoprotective effects of (+)-CLA with inhibiting ferroptosis.

### (+)-CLA suppresses APAP-induced and erastin-induced hepatocyte ferroptosis in vitro

**Fig. 3:**
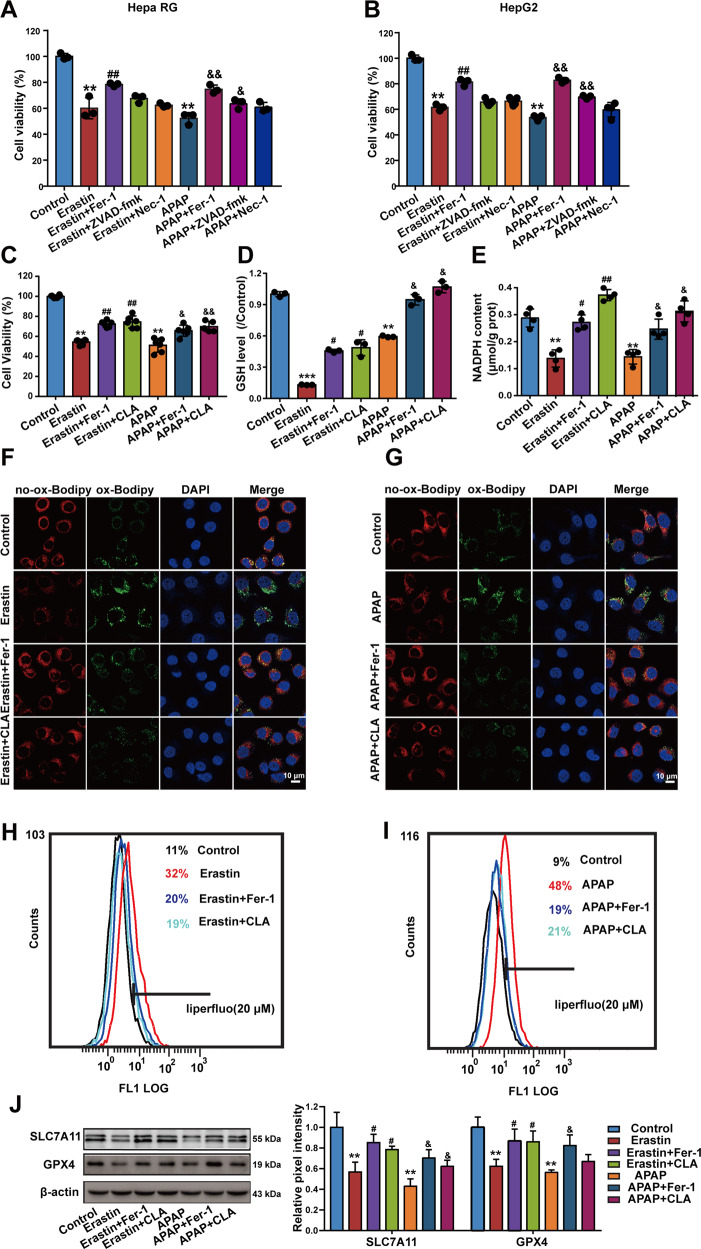
(+)-CLA suppresses APAP-induced and erastin-induced ferroptosis in vitro. **a**, **b** The effects of different inhibitors on APAP-induced or erastin-induced cytotoxicity. Hepa RG and HepG2 cells were pre-treated with or without ZVAD-fmk (10 μM), nec-1 (10 μM), or fer-1 (1 μM) for 1 h, and then treated with APAP (10 mM) or erastin (40 μM) for another 24 h. After treatment, MTT assay were performed to quantify role of apoptosis and necroptosis. **c**–**j** Hepa RG cells were pre-treated with (+)-CLA (5, 20 μM) and fer-1 (1 μM) for 1 h, and then treated with APAP (10 mM) and erastin (40 μM) for another 24 h. **c** Cell viability was measured by the MTT assay. **d** The GSH content in cells was measured using HPLC-ECD. **e** The NADPH level was determined by a commercial kit. **f**, **i** The accumulation of lipid peroxidation in cells was analyzed by BODIPY and liperfluo staining, followed by confocal imaging and flow cytometry analysis, respectively. Scale bar = 10 μm. **j** Protein expressions of SLC7A11 and GPX4 were analyzed by western blot (left panel) and the band intensities relative to β-actin were quantified using image J software (right panel). Data are expressed as mean ± SD of three independent experiments and the statistical differences were analyzed by one-way or two-way ANOVA (**a**–**c**, one-way ANOVA; **h**, two-way ANOVA). *P < 0.05, **P < 0.01, ***P < 0.001 vs. control group; ^#^*P* < 0.05, ^##^*P* < 0.01 vs. erastin group; ^&^*P* < 0.05, ^&&^*P* < 0.01 vs. APAP group.

To further obtain in vitro evidence of the involvement of ferroptosis in DILI, inhibitors including ZVAD-fmk, nec-1, and fer-1 were utilized to explore the forms of cell death occurred during APAP-induced or erastin-induced cell death. Data show that both ZVAD-fmk and fer-1 alleviated APAP-induced cell death to some extent, but only fer-1 protected against erastin-induced cell death (Fig. 3a, b and Figs. S6, S7). These results suggested that both ferroptosis and apoptosis were involved in APAP-induced cell death. Moreover, to certify the inhibitory activity of (+)-CLA on ferroptosis, we assessed the sensitivities of different hepatic cell lines (Hepa RG, SMMC-7721, HepaG2, and Bel-7402) to the ferroptosis inducer RSL3, a specific GPX4 inhibitor. Comparing with other cell lines, Hepa RG was the most sensitive to RSL3, with an IC_50_ of 3.7 µM (Fig. S4). APAP and erastin also induced significant cytotoxicity in Hepa RG, with IC_50_ of 9.8 mM and 39 µM, respectively (Fig. S5). Thus, this cell line was chosen to confirm the effects of (+)-CLA on ferroptosis in vitro. Results found that both fer-1 and (+)-CLA somewhat ameliorated APAP-induced or erastin-induced cytotoxicity (Fig. 3c and Fig. S8), and prevented the depletion of GSH and NADPH in Hepa RG (Fig. 3d, e). When monitoring lipid peroxidation using C11-BODIPY and liperfluo, we found that (+)-CLA significantly reduced the accumulation of lipid peroxidation induced by APAP or erastin in Hepa RG cells (Fig. 3f–i). Furthermore, we discovered that (+)-CLA upregulated the protein levels of SLC7A11 and GPX4 in Hepa RG cells treated with APAP or erastin (Fig. 3j). These in vitro data further support the idea that anti-ferroptosis is involved in the molecular mechanism of (+)-CLA against DILI.

### (+)-CLA attenuates APAP-induced cytotoxicity through activating the Keap1–Nrf2 pathway

**Fig. 4:**
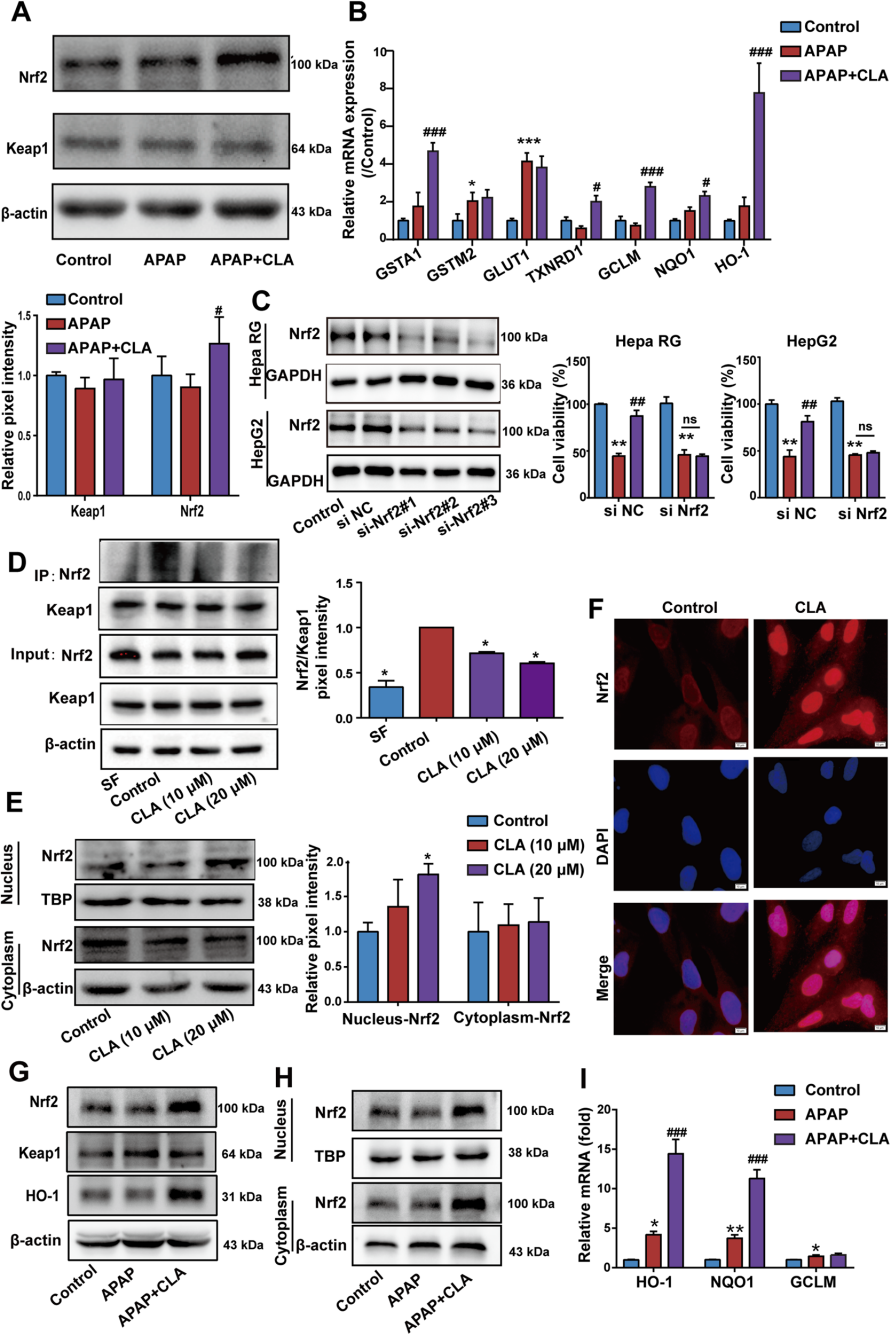
(+)-CLA attenuates APAP-induced cytotoxicity through the activation of Keap1–Nrf2 pathway. **a**, **b** Hepa RG cells were pre-incubated with (+)-CLA (5 μM) for 1 h, and then incubated with APAP (10 mM) for another 24 h. **a** The protein expressions of Keap1 and Nrf2 were analyzed by western blot (upper panel) and the band intensities relative to β-actin were quantified using image J software (bottom panel). **b** The mRNA level of Nrf2 was analyzed by qRT-PCR. **c** The influence of Nrf2 on the protective effect of (+)-CLA on APAP-induced cytotoxicity was determined. After transfaction with control siRNA (no silencing) or Nrf2 siRNA for 6 h, cells were incubated with (+)-CLA (5 μM) for 1 h, and then treated with APAP (10 mM) for another 24 h. The cell viability was measured by the MTT assay. **d** The interaction between Keap1 and Nrf2 was determined by Co-IP assay. HepG2 cells, transfected with Nrf2 and Keap1 plasmids, were treated with or without (+)-CLA for 6 h, and immunoprecipitated proteins were subjected to western blot analysis with Nrf2 and Keap1 antibodies. **e**, **f** (+)-CLA promoted the nuclear translocation of Nrf2. Cells were incubated with or without indicated doses of (+)-CLA for 18 h. The protein levels of Nrf2 in the nucleus and cytoplasm were determined using **e** western blot analysis and **f** immunofluorescence. Scale bar = 10 μm. **g**–**i** The effect of (+)-CLA on Keap1–Nrf2 pathway was investigated in the liver tissues of APAP-treated mice. **g** The protein expressions of HO-1, Keap1, and Nrf2 were analyzed by western blot. **h** The protein expressions of Nrf2 in the nucleus and cytoplasm were analyzed by western blot. **i** The mRNA levels of HO-1, NQO1, and GCLM were analyzed by qRT-PCR. Data are expressed as mean ± SD of three independent experiments and the statistical differences were analyzed by one-way or two-way ANOVA (**a**–**c**, **e** and **i**, two-way ANOVA; **d** one-way ANOVA). *P < 0.05, **P < 0.01, ***P < 0.001 vs. control group; ^#^*P* < 0.05, ^##^*P* < 0.01, ^###^*P* < 0.001 vs. APAP group.

It has been revealed that Keap1–Nrf2 plays a central role in protecting hepatocellular carcinoma (HCC) cells against ferroptosis^10^. Cumulative evidence indicated that several natural molecules exerted hepatoprotective effects through activating the Keap1–Nrf2 pathway, which implied that the activation of this pathway might protect against erastin-induced ferroptosis in hepatocellular carcinoma (HCC) cells^28,29^. Therefore, we hypothesized that the hepatoprotection mechanism of (+)-CLA was associated with regulating the Keap1–Nrf2 pathway. To prove this hypothesis, we firstly examined the influence of (+)-CLA on Nrf2 protein expression and its downregulated factors, such as HO-1, NQO1, and GCLM^30^, in APAP-treated Hepa RG cells. As shown in Fig. 4a, b, treatment with (+)-CLA in the presence of APAP enhanced the expression of Nrf2 protein and its downstream target genes including GSTA1, TXNRD1, GLCM, NQO1, and HO-1. Subsequently, knockdown of Nrf2 by specific siRNA in Hepa RG and HepG2 cells was used to further evaluate the protective effect of (+)-CLA against APAP-induced cytotoxicity and the dependence of this protection on Nrf2 activation in vitro. The results of western blot showed an obvious reduced Nrf2 expression in cells transfected with Nrf2 siRNA (Fig. 4c) Co-treatment with (+)-CLA and APAP clearly improved cell survival compared with APAP treatment alone. However, (+)-CLA-induced protective effects were diminished when Nrf2 was knocked down (Fig. 4c).

In stressed conditions, Nrf2 releases from its repressor Keap1, and translocases into the nucleus to activate the transcription of cytoprotective genes. Thus, we examined the effects of (+)-CLA on the interaction between Keap1 and Nrf2, and the accumulation of nuclear Nrf2 in Hepa RG cells. Result from Co-IP assay demonstrated that the Keap1–Nrf2 interaction was interrupted in the presence of (+)-CLA (Fig. 4d). Moreover, western blot analysis indicated that the protein levels of Nrf2 in the nucleus were increased in cells treated by (+)-CLA (Fig. 4e), which was verified by the accumulation of Nrf2 in the nucleus via IF (Fig. 4f).

We further verified the impact of (+)-CLA on Keap1–Nrf2 pathway in vivo. Data in Fig. 4g show that (+)-CLA increased the protein expressions of Nrf2 and HO-1, but had no effect on protein expression of Keap1 in the live tissues of APAP-treated mice. Moreover, (+)-CLA was found to enhance the expression of nuclear Nrf2 and its downstream target genes including HO-1, NQO1, and GCLM (Fig. 4h, i). Collectively, the results obtained from in vivo and in vitro experiments suggest that the activation of the Keap1–Nrf2 pathway by (+)-CLA contributes to its protective effect against DILI.

### (+)-CLA targets Keap1–Cys151 to inhibit Nrf2 ubiquitylation

**Fig. 5:**
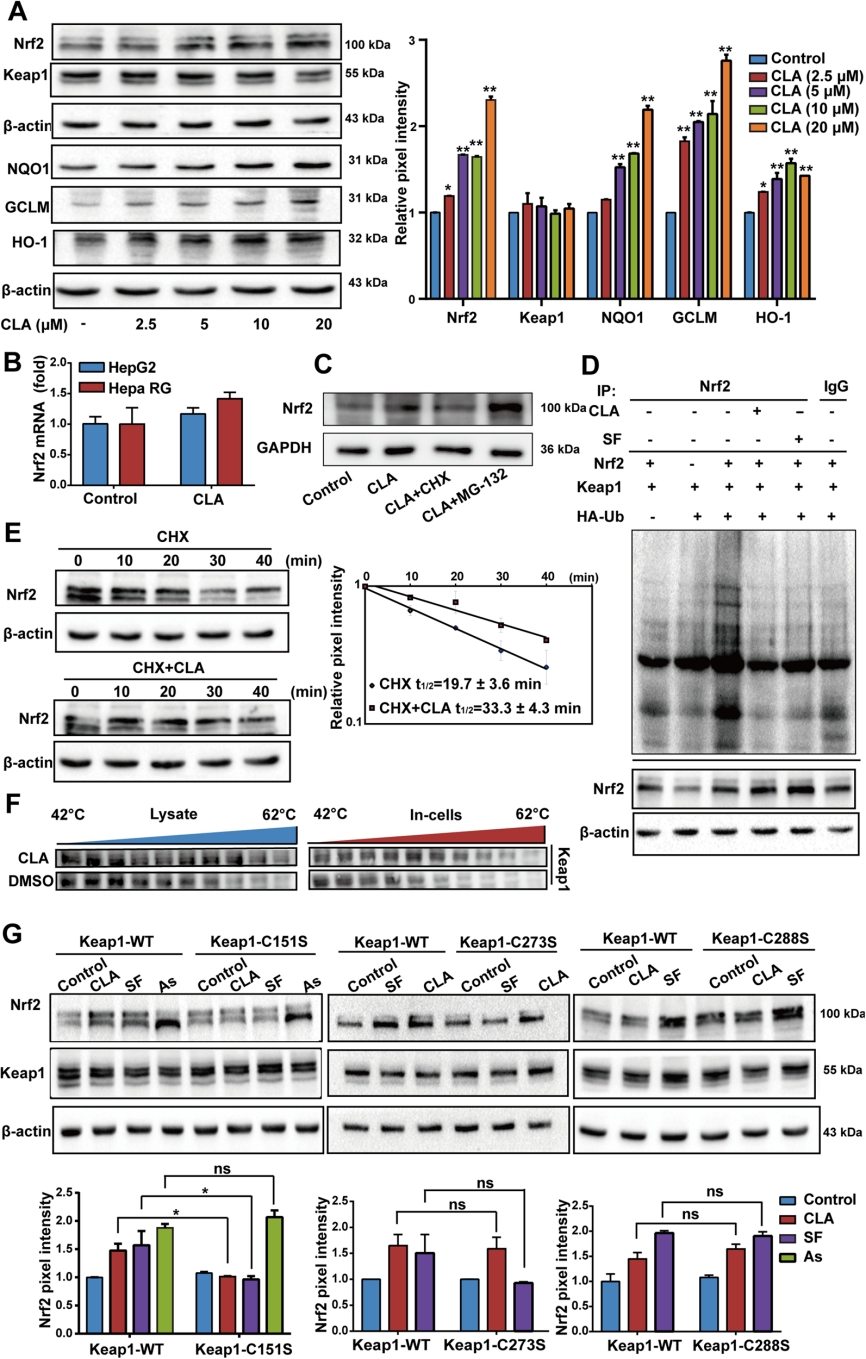
(+)-CLA targets Keap1–Cys151 to inhibit Nrf2 ubiquitylation and renders Nrf2 activation. **a** The protein expressions of Keap1, Nrf2, NQO1, γ-GCS, and HO-1 were determined in Hepa RG cells treated with or without the indicated doses of (+)-CLA for 18 h. **b** Nrf2 mRNA level was analyzed by qRT-PCR in cells treated with or without (+)-CLA (20 μM, 24 h). **c** CHX limited whereas MG-132 augmented the increase in Nrf2 protein levels caused by (+)-CLA. Cells were treated with (+)-CLA (20 μM) with or without CHX (20 μg/ml) or MG-132 (5 μM) for 24 h and Nrf2 protein levels were assayed by western blot. **d** (+)-CLA blocked the ubiquitylation of Nrf2. HepG2 cells, co-transfected with plasmids encoding HA-ubiquitin, Nrf2, and Keap1, were exposed to MG-132 (10 μM) in the presence or absence of (+)-CLA (10 μM) for 6 h. Anti-Nrf2 immunoprecipitates were subjected to western blot analysis with anti-HA antibody for the detection of ubiquitylated Nrf2. SF (2 μM) was used as a positive control. **e** (+)-CLA increased the half-life of Nrf2. Cells were pre-incubated with or without (+)-CLA for 8 h. After exposure to 50 μM CHX, cells were lysed at the indicated time point and then western blot analysis was used to analyze protein levels of Nrf2. **f** (+)-CLA enhanced the thermal stability of Keap1. Keap1 protein stability was measured by cellular thermal shift assay in the cell lysate or intact cells, which were exposed to (+)-CLA (20 μM). **g** (+)-CLA activated the Keap1–Nrf2 pathway in a Keap1–Cys-151-dependent manner. Cells were transfected with Keap1–siRNA that targeted the 5′ untranslated region to knock down endogenous Keap1. Concurrently, Keap1 wild-type (Keap1-WT), Cys151-mutated Keap1 (Keap1–C151S), Cys273-mutated Keap1 (Keap1–C273S) or Cys288-mutated Keap1 (Keap1–C288S) were individually transfected into these cells, and the cells were exposed to (+)-CLA, SF, or As (III) for 16 h. The expressions of Keap1 and Nrf2 were analyzed by western blot. Data are expressed as mean ± SD of three independent experiments and the statistical differences were analyzed by two-way ANOVA or *t*-tests (**a**, **g** two-way ANOVA; **b** *t*-tests). *P < 0.05, **P < 0.01 vs. control group.

To further characterize the mechanism of Nrf2 activation by (+)-CLA, we assessed the protein and mRNA levels of Nrf2 in HCC cells treated with (+)-CLA. Of note, (+)-CLA (20 μM) significantly enhanced Nrf2 protein expression (Fig. 5a) but had no impact on its mRNA level (Fig. 5b), suggesting that (+)-CLA upregulated the Nrf2 protein level in a transcription-independent manner. Next, CHX (a protein synthesis inhibitor) and MG-132 (a selective 26S proteosomal inhibitor) were used to determine if (+)-CLA regulates the protein stability of Nrf2. As shown in Fig. 5c, no difference in Nrf2 protein levels was observed in cells treated with (+)-CLA in the presence or absence of CHX. However, co-treatment with MG-132 and (+)-CLA greatly augmented (+)-CLA-induced elevation of Nrf2 protein levels (Fig. 5c), indicating that (+)-CLA activated Nrf2 by blocking its protein degradation rather than its synthesis. To confirm this assumption, we tested the effect of (+)-CLA on regulating Nrf2 ubiquitylation since Keap1-mediated Nrf2 ubiquitylation is essential for its 26S proteasome-mediated degradation. Our results showed that (+)-CLA blocked the ubiquitylation of Nrf2 in a manner similar to sulforaphane (SF, a well-known Nrf2 activator) (Fig. 5d). Further, we measured the half-life of Nrf2 in the presence or absence of (+)-CLA. As expected, (+)-CLA prolonged the half-life of Nrf2 (Fig. 5e). Thus, these results indicate that (+)-CLA activates the Keap1–Nrf2 signaling pathway through inhibiting Nrf2 ubiquitylation and thereby increases Nrf2 protein stability.

It has been reported that Nrf2 activators can bind to Keap1 and induce conformational changes in Keap1. This may block ubiquitin-mediated Nrf2 degradation in the cytosol, leading to subsequent nuclear translocation of Nrf2^31^. Thus, we investigated the potential capability of (+)-CLA on binding to the Keap1 protein using a cellular thermal shift assay. As observed in Fig. 5f, (+)-CLA treatment efficiently protected Keap1 from temperature-dependent degradation. Keap1 is a cysteine-rich protein containing many highly reactive sulfhydryl groups, which facilitate its direct binding with small molecules and affect the activation of Nrf2^32^. Herein, the possibility of (+)-CLA to form covalent adducts with high-reactive cysteines including Cys151, Cys273, and Cys288 of Keap1 in HCC cells was investigated. Cells were transfected with Keap1–siRNA to silence endogenous Keap1 protein, and reconstituted with plasmids expressing Keap1-WT, Cys151-mutated Keap1 (Keap1–Cys151S), Cys273-mutated Keap1 (Keap1–Cys273S) or Cys288-mutated Keap1 (Keap1–Cys288S). We found that the (+)-CLA-induced upregulation of Nrf2 protein levels were significantly abrogated in cells transfected with Keap1–Cys151S, but not Keap1–Cys273S or Keap1–Cys288S (Fig. 5g). This data suggests that Cys151 in Keap1 is essential for (+)-CLA-induced activation of Nrf2.

## Discussion

An overdose of APAP is a well-known detrimental factor for DILI. NAC is the most common clinically used therapeutic agent for APAP detoxification. However, its effectiveness is limited to the early stages of DILI and it often causes side effects such as vomiting, nausea, and even shock^33–35^. Therefore, it is essential to elucidate the mechanism of APAP-induced hepatotoxicity and develop additional drugs for the treatment of DILI. Previous reports demonstrated that apoptosis is involve in APAP-induced hepatocyte death, which has been confirmed in our study (Fig. 3a, b). The current study reports another innovative cell death mode, namely ferroptosis also contribute to DILI. Two major findings support such conclusion. Firstly, APAP induces an obvious hepatotoxicity in a similar pattern to erastin (a ferroptosis inducer), and the specific ferroptosis inhibitor fer-1 confers a hepatoprotection against both APAP-induced and erastin-induced liver injury. Secondly, (+)-CLA prevents liver from DILI by suppressing hepatocytes ferroptosis. Mechanistically, this natural molecule activates the Keap1–Nrf2 pathway in a Keap1–Cys151-dependent manner. By contrast, iron chelators and specific lipid peroxidation scavengers can prevent ferroptosis^7^.

Since the discovery of ferroptosis in 2012^36^, the mechanisms underlying ferroptosis have been gradually disclosed. Prominently, iron-dependent accumulation of lipid peroxidation is deemed as the lethal factor^7^. Ferroptosis can be triggered once the balance between the generation and the clearance of lipid peroxidation is disrupted, such as the inhibition of GPX4 and the depletion of GSH^25^. GPX4, with the help of GSH, converts toxic lipid hydroperoxides into nontoxic lipids^8^. In our study, cell death and a robust production of lipid peroxidation have been concomitantly observed in APAP-treated or erastin-treated mice and hepatocytes, accompanied with reduced GPX4 activity and decreased GSH content. This evidence sufficiently proves our hypothesis that ferroptosis is engaged in the mechanism of APAP-caused DILI. In fact, our findings are supported by previous reports delineating mechanisms underlying DILI. On one hand, it has been indicated that an overdose of APAP increased the oxidative metabolism of P450 2E1 to induce the accumulation of a large amount of N-acetyl-p-benzoquinone imine, consequently depleting GSH and causing ROS accumulation in hepatocytes^37^. On the other hand, iron, which was formerly deemed as a catalyst for normal ROS production through the Fenton reaction^38–40^, now is recognized as an initiator of lipid peroxides and plays an on–off switching role in ferroptosis^23^. Studies in vivo showed that iron overload caused liver injury in rats and mice^38,41^. Moreover, clinical research has demonstrated that increased hepatic iron stores and elevated serum ferritin concentrations are common features of various liver diseases^42,43^. Of note, in APAP-induced hepatotoxicity, lysosomal iron was revealed to transfer into the mitochondria and promote oxidation^44,45^, while this detrimental effect was antagonized by iron chelators^46^. In consistent with these earlier studies, our results explicitly define the role of ferroptosis in APAP-induced DILI, which shed significant light on the improvement of therapies for liver diseases.

In our search for anti-DILI compounds, (+)-CLA aroused our attention since it improves chemical-induced liver injury and increases GSH content^21,22^. Intriguingly, our study revealed that (+)-CLA alleviated both APAP-induced and erastin-induced hepatotoxicity as effectively as fer-1 in vivo and in vitro. Next, we tested the effect of (+)-CLA on liver antioxidant systems and found that it significantly attenuated APAP or erastin provoked lipid peroxidation, manifested by the levels of MDA, 4-HNE and lipid ROS in liver tissues and hepatocytes. In addition, (+)-CLA also abolished other ferroptosis markers raised by APAP or erastin treatment, including the decreased expressions of GPX4, SLC7A11, and Ptgs2, the reduced activity of GPX4, as well as the depletions of GSH and NADPH. Thus, we conclude that (+)-CLA protects against APAP-induced liver injury through inhibiting hepatocyte ferroptosis.

The Nrf2-mediated defensive response can effectively protect cells and tissues against toxins, oxidants, and drugs^47^. Inhibition of the Nrf2 pathway has been reported to increase ferroptosis sensitivity^48^. Several chemical Nrf2 activators have demonstrated the protective effects against APAP-induced hepatotoxicity^49,50^. A previous study has indicated that Nrf2 protects cells from ferroptosis by increasing GSH production^14^. Here, we showed that treatment with (+)-CLA increased the protein levels of GCLM, which is a Nrf2-target gene and a key enzyme for GSH synthesis, and accordingly increased intracellular GSH levels^50^. Under normal conditions, the repressor protein Keap1 forms an inactive complex with Nrf2 and promotes the degradation of Nrf2 by the ubiquitin-proteasome pathway^51^. Upon stimulation by electrophiles and antioxidants, Nrf2 dissociates from Keap1, translocates into the nucleus to bind with ARE, and subsequently promotes the transcriptions of antioxidant and antiferroptosis-related genes^52^. Our current study discovers that (+)-CLA directly binds with Keap1 to facilitate the blockage of ubiquitination-related Nrf2 degradation, and thereby activates Nrf2 nuclear translocation. Among the cysteine residues in Keap1, Cys-151, Cys-273, and Cys-288 are sensor sites that are particularly active for reacting with potential Nrf2 activators^53,54^. We have identified Cys-151, but not Cys-273 or Cys-288, as a binding site of Keap1 for (+)-CLA, which is a similar pattern to SF^55^. These results allow us to present a working model for the mechanism of Nrf2 activation by (+)-CLA. In this model, (+)-CLA specifically modifies the Cys151 residue of Keap1 to interrupt the Keap1–Nrf2 interaction, through which it blocks the proteasome-mediated degradation of Nrf2 and promotes its nuclear translocation followed by the transcriptions of cytoprotective genes.

## Conclusion

Our current study provides the first evidence that (+)-CLA protects against APAP-induced DILI via inhibiting ferroptosis and activating the Keap1–Nrf2 pathway in a Cys-151-dependent manner. These results establish a framework for the development of innovative strategies to treat DILI, especially those induced by APAP overdose in clinic.

## Supplementary information

Fig.S1

Fig.S2

Fig.S3

Fig.S4

Fig.S5

Fig.S6

Fig.S7

Fig.S8

Supplementary figure legends

supplementary table
