# Supplementary figures and images for: (+)-Clausenamide protects against drug-induced liver injury by inhibiting hepatocyte ferroptosis
Source: Cell Death Dis. 2020 Sep 19;11(9):781. doi: 10.1038/s41419-020-02961-5 (PMC7502081; doi:10.1038/s41419-020-02961-5)

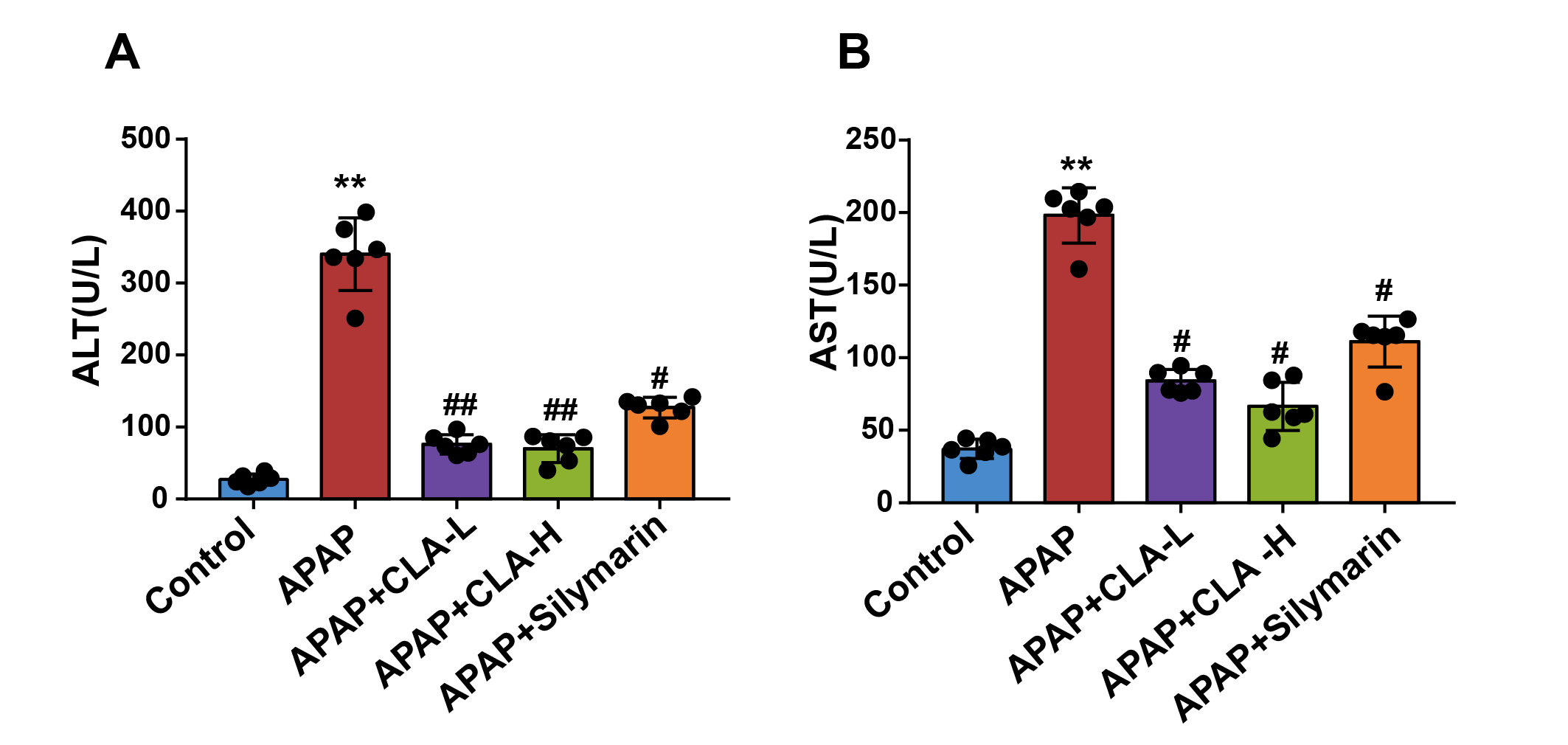

Supplement: Supplementary file 1 — Fig.S1 [file 41419_2020_2961_MOESM1_ESM.png]

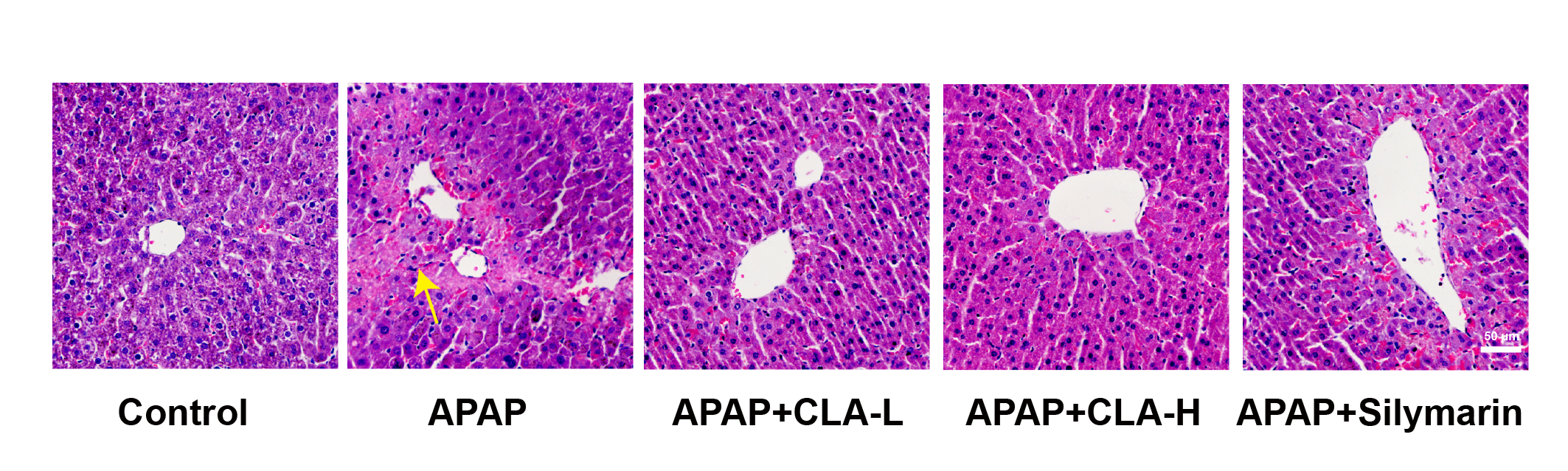

Supplement: Supplementary file 2 — Fig.S2 [file 41419_2020_2961_MOESM2_ESM.png]

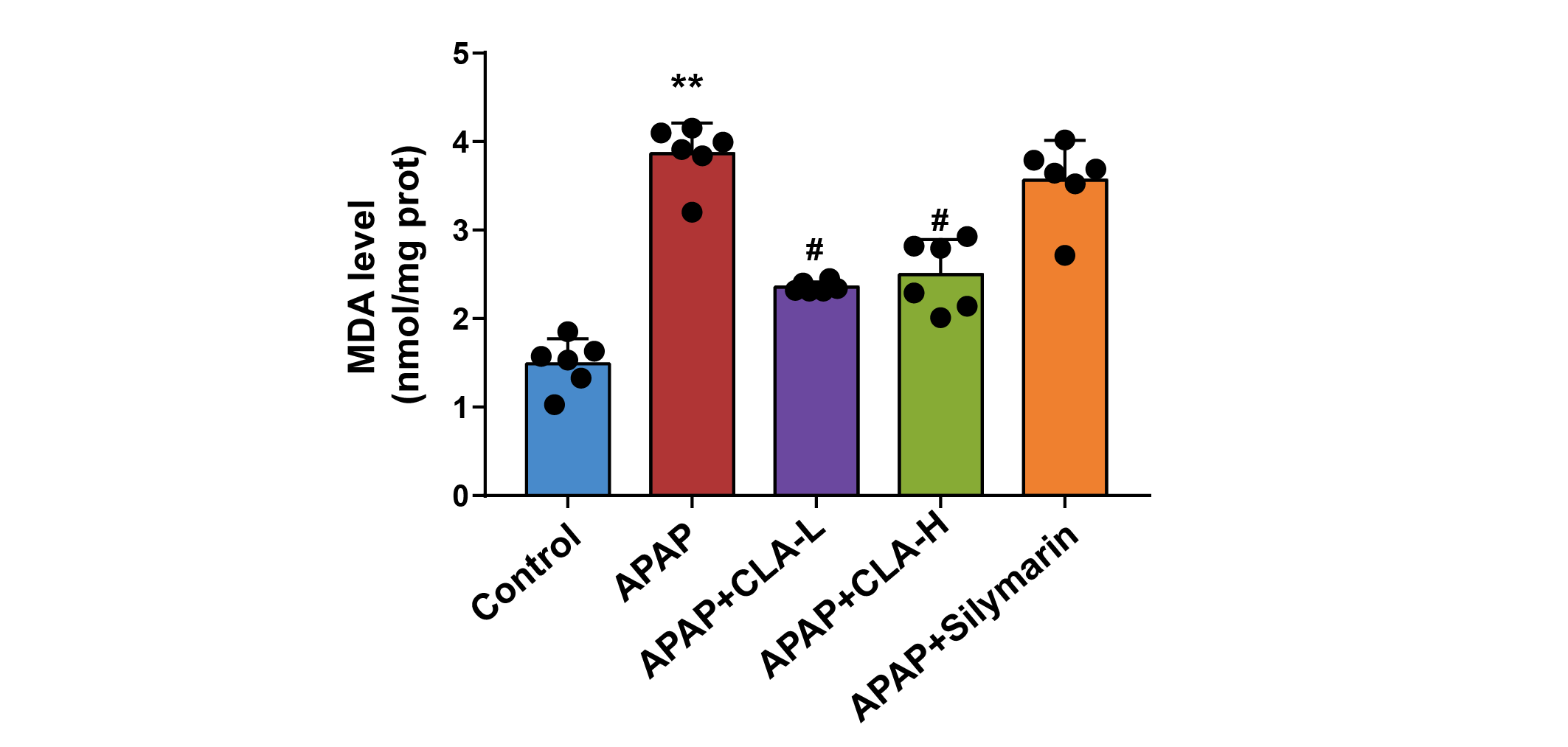

Supplement: Supplementary file 3 — Fig.S3 [file 41419_2020_2961_MOESM3_ESM.png]

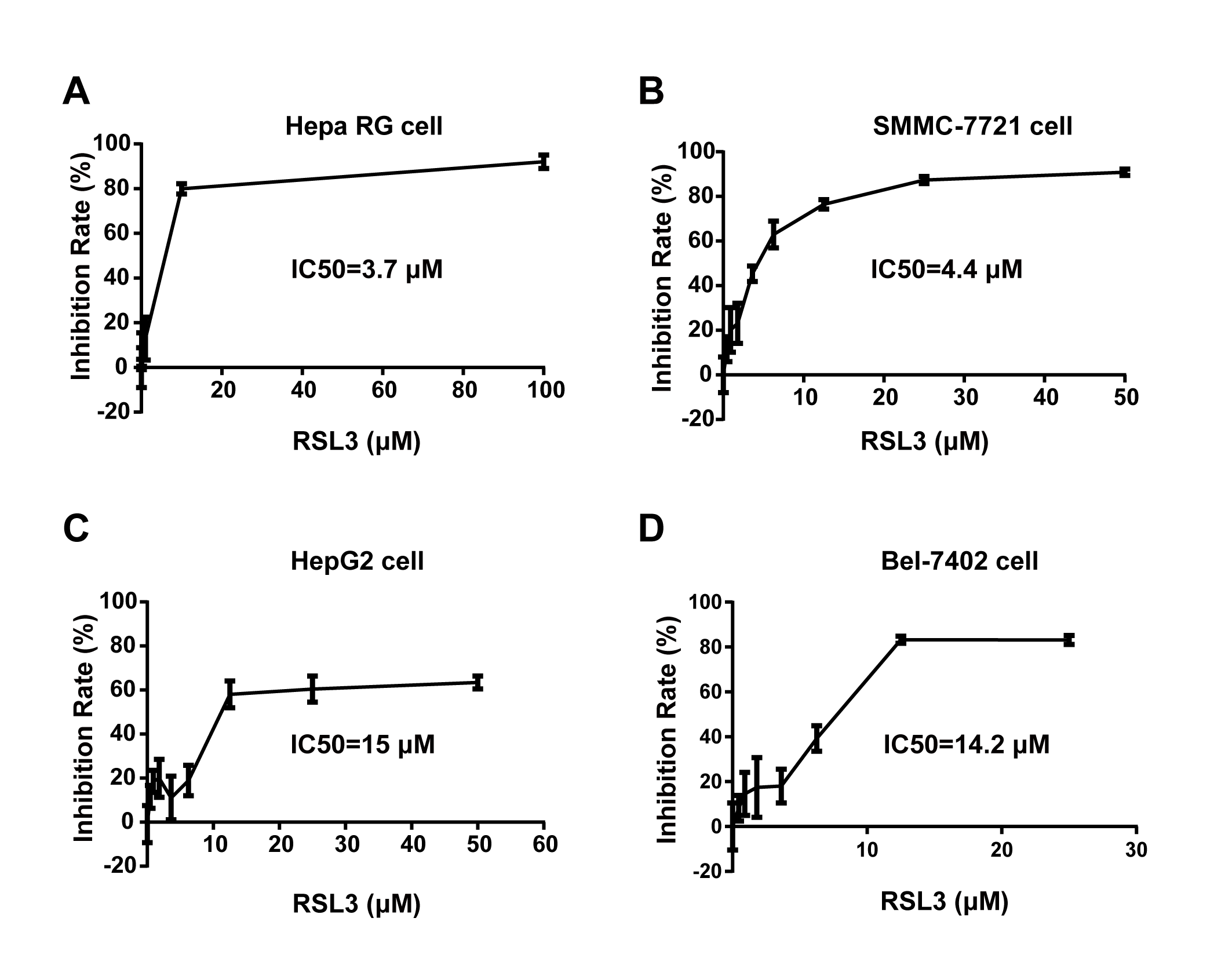

Supplement: Supplementary file 4 — Fig.S4 [file 41419_2020_2961_MOESM4_ESM.png]

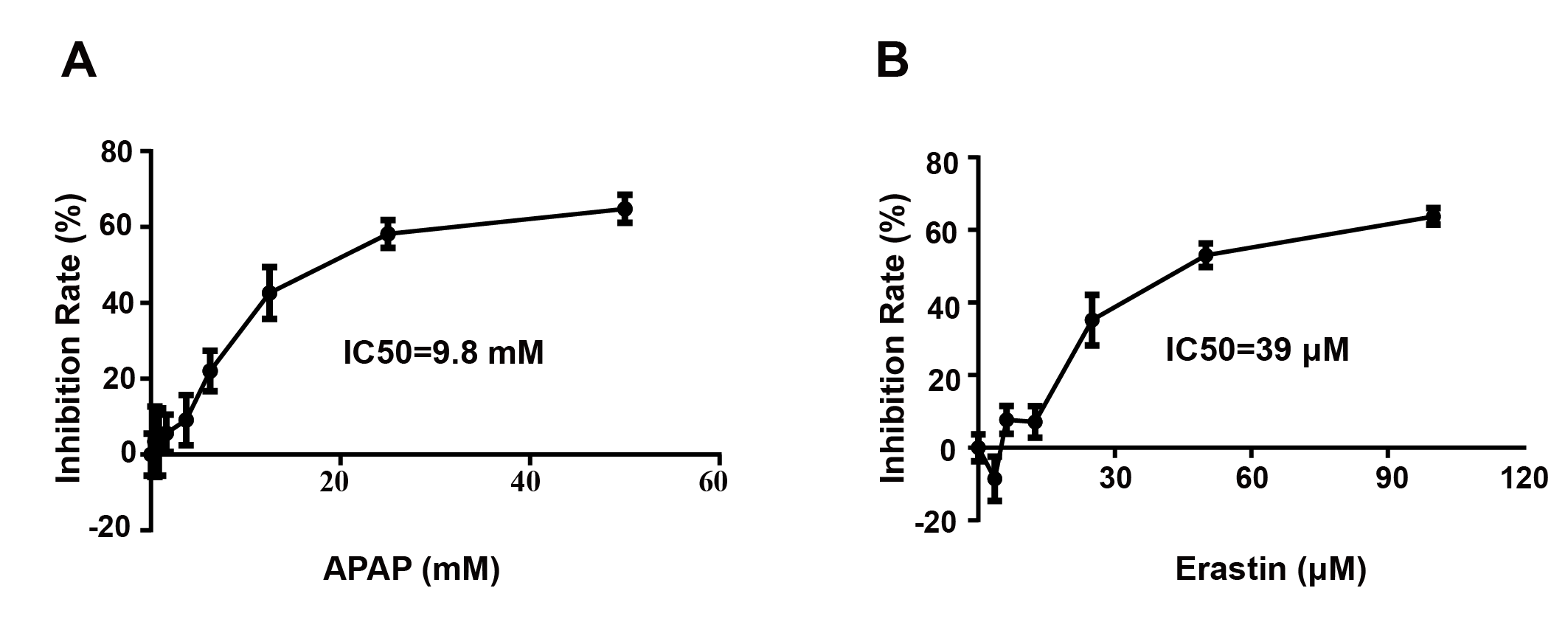

Supplement: Supplementary file 5 — Fig.S5 [file 41419_2020_2961_MOESM5_ESM.png]

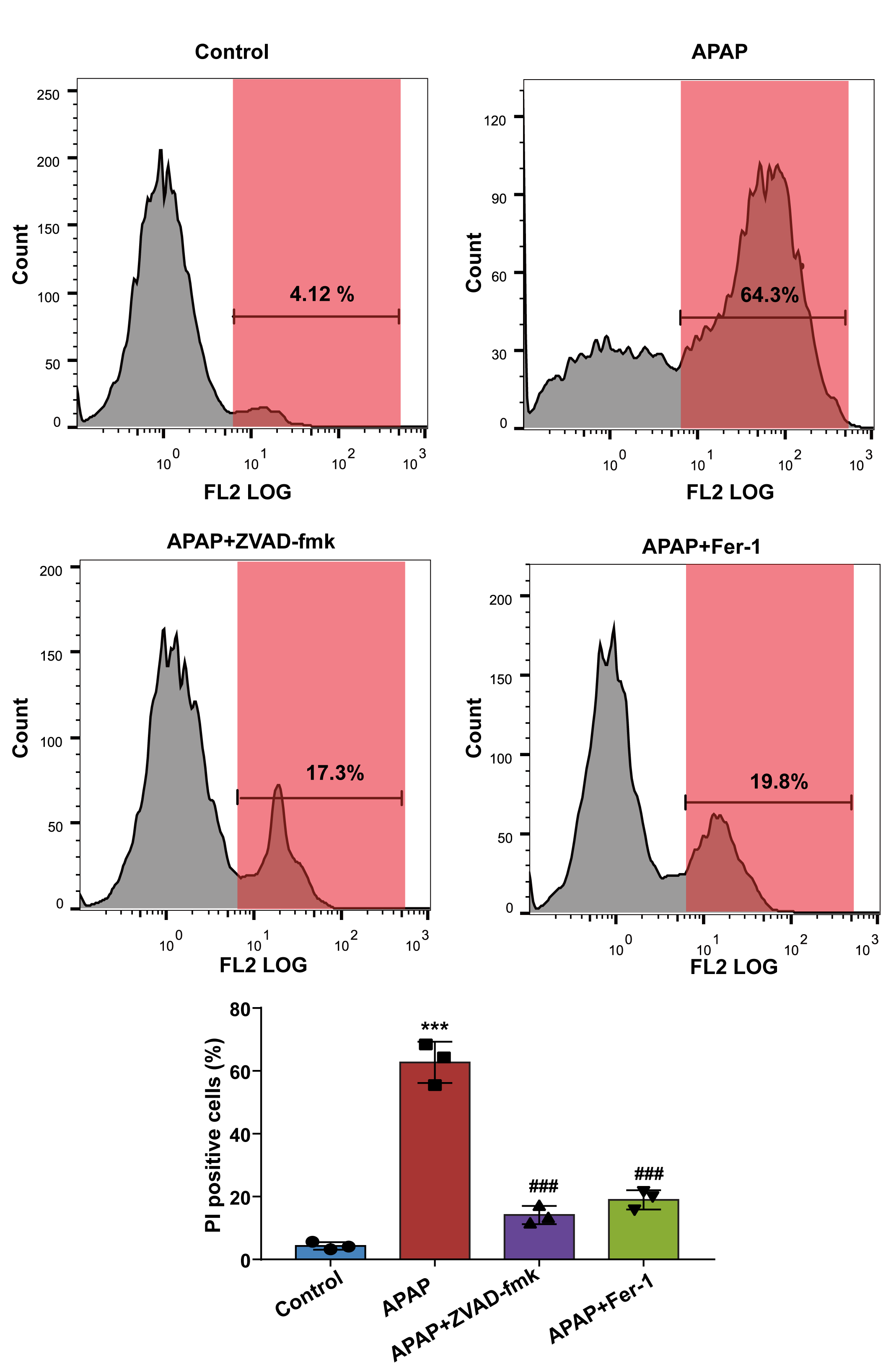

Supplement: Supplementary file 6 — Fig.S6 [file 41419_2020_2961_MOESM6_ESM.png]

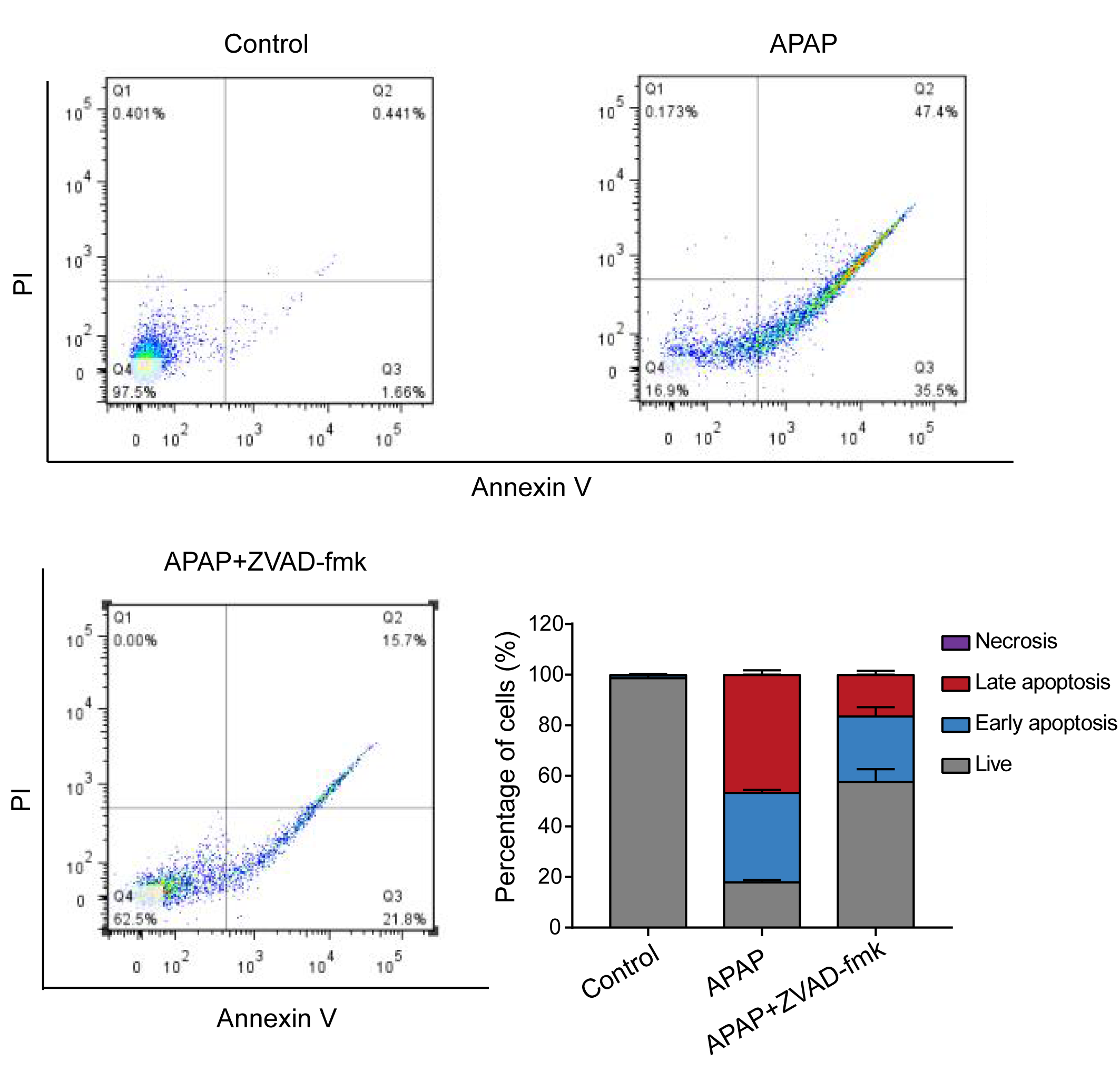

Supplement: Supplementary file 7 — Fig.S7 [file 41419_2020_2961_MOESM7_ESM.png]

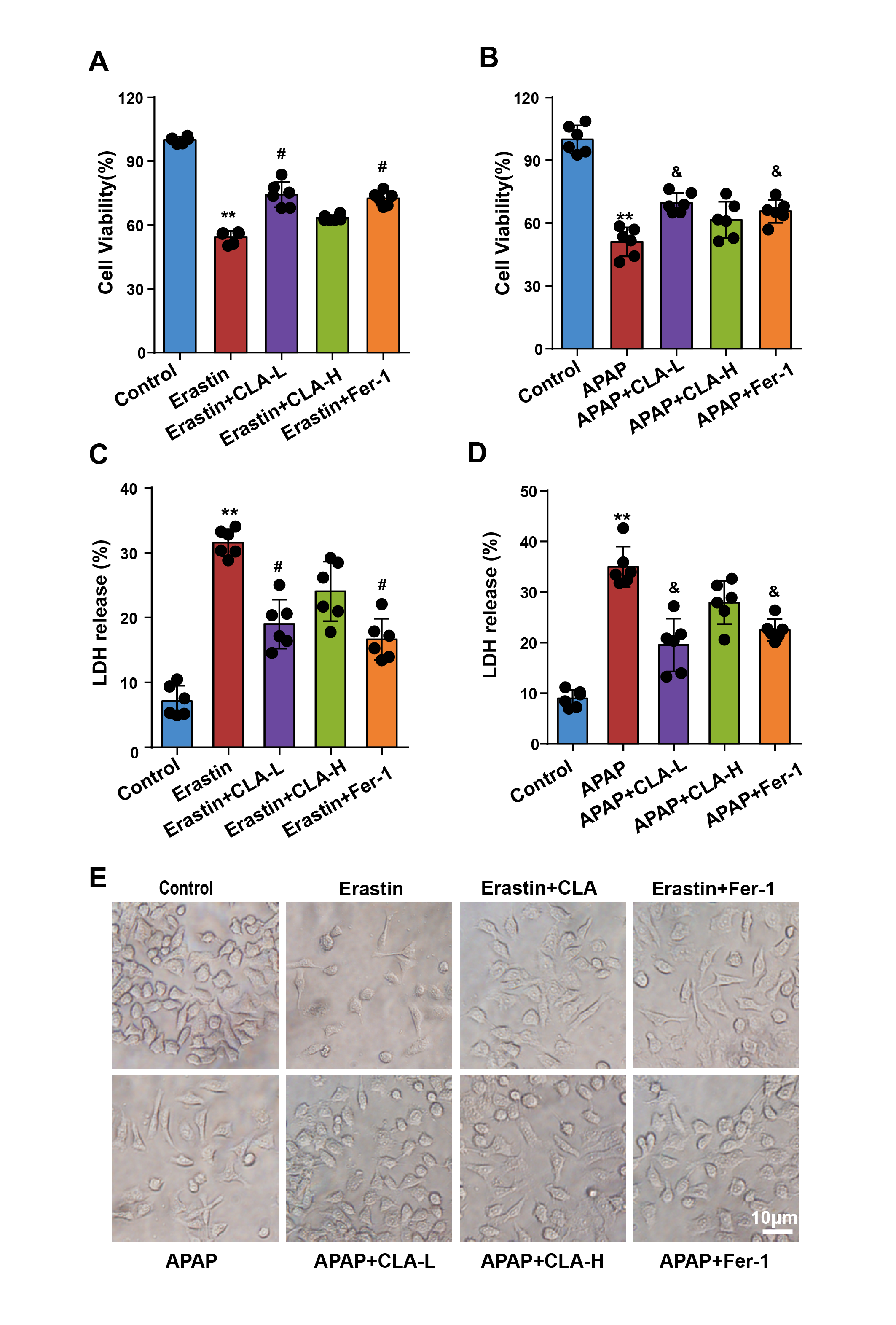

Supplement: Supplementary file 8 — Fig.S8 [file 41419_2020_2961_MOESM8_ESM.png]
