# Supplementary material for: (+)-Clausenamide protects against drug-induced liver injury by inhibiting hepatocyte ferroptosis
Source: Cell Death Dis. 2020 Sep 19;11(9):781. doi: 10.1038/s41419-020-02961-5 (PMC7502081; doi:10.1038/s41419-020-02961-5)
Supplement: Supplementary file 9 — Supplementary figure legends [file 41419_2020_2961_MOESM9_ESM.docx]

**Supplementary Fig. S1.** Serum levels of ALT and AST was detected by commercial assay kits. Mice were pretreated with (+)-CLA (i.g., 50, 100 mg/ kg) for 7 consecutive days. On the last day, mice were injected with a single dose of APAP (i.p., 600 mg/kg) at 1 h after administration of (+)-CLA. Silymarin (i.g., 100 mg/kg) was used as positive control. Serum ALT or AST activities were detected using ALT and AST kits, respectively. Data are expressed as means ± SD and the statistical differences were analyzed by one-way ANOVA (n = 6). ^**^P <0.01 vs. control group, ^#^P < 0.05, ^##^P < 0.01 vs. APAP group.

**Supplementary Fig. S2.** Histopathological changes were examined by H&E staining and observed under microscopy. The yellow and green arrows indicate bleeding and inflammatory infiltration, respectively. scale = 50 μm.

**Supplementary Fig. S3.** The content of hepatic MDA was measured using the MDA assay kit. Data are expressed as means ± SD and the statistical differences were analyzed by one-way ANOVA (n = 6). ^**^P <0.01 vs. control group, ^#^P < 0.05 vs. APAP group.

**Supplementary Fig. S4.** Cytotoxicity of RSL3 in different hepatic cell lines. Different hepatic cell lines were treated with indicated concentration of RSL3 for 24 h, and then the inhibitory rate was determined by MTT assay. (A) Hepa RG cell, (B) SMMC-7721 cell, (C) HepaG2 cell, (D) Bel-7402 cell. Results are expressed as means ± SD of 3 independent experiments.

**Supplementary Fig. S5.** Cytotoxicity of APAP and erastin in Hepa RG cells. Cells were treated with indicated concentration of APAP or erastin for 24 h, and then inhibitory rate was subjected to MTT assay. (A) The cytotoxicity of APAP, (B) The cytotoxicity of erastin. Results were expressed as means ± SD of 3 independent experiments.

**Supplementary Fig. S6.** The effect of different inhibitors on APAP-induced cell death. Hepa RG cells were pre-treated with or without ZVAD-fmk (10 μM) or fer-1 (1 μM) for 1 h, and then treated with APAP (10 mM) for another 24 h. After treatment, to quantify dead cells, cell samples were analyzed by a flow cytometry. Data are expressed as means ± SD and the statistical differences were analyzed by one-way ANOVA (n = 3). ^***^P <0.001 vs. control group, ^###^P < 0.001 vs. APAP group.

**Supplementary Fig. S7.** APAP induced apoptotic cell death in HepaRG cells. Hepa RG cells were pre-treated with or without ZVAD-fmk (10 μM) for 1 h, and then treated with APAP (10 mM) for another 24 h. After treatment, apoptotic cell death of treated cells was detected by dual staining with Annexin V-FITC and PI followed by flow cytometric analysis. The percentage of the following cell populations is indicated: Annexin V-FITC and PI negative stained, indicating viable cells (lower left quadrant), Annexin V-positive and PI-negative stained, indicating early apoptotic cells (lower right quadrant), and Annexin V/PI double-stained cells showing late apoptosis (upper right quadrant). Data are expressed as means ± SD and the statistical differences were analyzed by one-way ANOVA (n = 3).

**Supplementary Fig. S8.** The effect of (+)-CLA on APAP (B and D) and erastin (A and C) induced HepaRG cell injury and cell morphology (E). Hepa RG cells were pre-treated with (+)-CLA (5 μM, 20 μM) and fer-1 (1 μM) for 1 h, and then treated with APAP (10 mM) and erastin (40 μM) for another 24 h. Cell viability (A and B) were measured by MTT assay, the ratio of LDH release (C and D) were measured by LDH assay kit. Data are expressed as means ± SD and the statistical differences were analyzed by one-way ANOVA (n = 6). ^**^P <0.01 vs. control group, ^#^P < 0.05 vs. erastin group, ^&^P < 0.05 vs. APAP group.
