# Supplementary material for: (+)-Clausenamide protects against drug-induced liver injury by inhibiting hepatocyte ferroptosis
Source: Cell Death Dis. 2020 Sep 19;11(9):781. doi: 10.1038/s41419-020-02961-5 (PMC7502081; doi:10.1038/s41419-020-02961-5)
Supplement: Supplementary file 10 — supplementary table [file 41419_2020_2961_MOESM10_ESM.docx]

**(+)-Clausenamide protects against drug-induced liver injury by inhibiting hepatocyte ferroptosis**

| Species | Target | Forward primer (5'->3') | Reverse primer (5'->3') |
| --- | --- | --- | --- |
| Mice | Ptgs2 | CCTCGTCCAGATGCTA | CTCGGCTTCCAGTATTG |
| Mice | NQO1 | AGGATGGGAGGTACTCGAATC | TGCTAGAGATGACTCGGAAGG |
| Mice | GLCM | AGGAGCTTCGGGACTGTATCC | GGAAACTCCCTGACTAAATCG |
| Mice | HO-1 | AGGTACACATCCAAGCCGAGA | CATCACCAGCTTAAAGCCTTCT |
| Homo | GSTA1 | GACATTCATCTGGTGGAACT | GGTTGCTGATTCTGGTTTT |
| Homo | GSTM2 | GTAGGGCAGATTGGGAAA | GGGGACGCTCCTGATTAT |
| Homo | HO-1 | CCAGGCAGAGAATGCTGAGTTC | AAGACTGGGCTCTCCTTGTTGC |
| Homo | TXNRD1 | TCTGAGTCGGCCTGGTGT | CCCTGGTTGTTGGAGCAT |
| Homo | Nrf2 | CACATCCAGTCAGAAACCAGTGG | GGAATGTCTGCGCCAAAAGCTG |
| Homo | NQO1 | TGGCTAGGTATCATTCAACTC | CCTTAGGGCAGGTAGATTCAG |
| Homo | GCLM | ATCAAACTCTTCATCATCAAC | GATTAACTCCATCTTCAATAGG |
| Homo | β-actin | TTGCCGACAGGATGCAGAAGGA | AGGTGGACAGCGAGGCCAGGAT |
| Mice | 18s | GGGAGAGCGGGTAAGAGA | ACAGGACTAGGCGGAACA |

Supplementary Table 1. Primer pairs for RT-qPCR
